# Distributed Ledger Infrastructure to Verify Adverse Event Reporting (DeLIVER): Proposal for a Proof-of-Concept Study

**DOI:** 10.2196/28616

**Published:** 2021-06-10

**Authors:** Madison Milne-Ives, Ching Lam, Najib Rehman, Raja Sharif, Edward Meinert

**Affiliations:** 1 Centre for Health Technology University of Plymouth Plymouth United Kingdom; 2 Institute of Biomedical Engineering Department of Engineering Science University of Oxford Oxford United Kingdom; 3 FarmaTrust London United Kingdom; 4 Department of Primary Care and Public Health School of Public Health Imperial College London London United Kingdom

**Keywords:** adverse drug reaction reporting systems, drug-related side effects and adverse reactions, blockchain, mobile applications, distributed ledger technology

## Abstract

**Background:**

Adverse drug event reporting is critical for ensuring patient safety; however, numbers of reports have been declining. There is a need for a more user-friendly reporting system and for a means of verifying reports that have been filed.

**Objective:**

This project has 2 main objectives: (1) to identify the perceived benefits and barriers in the current reporting of adverse events by patients and health care providers and (2) to develop a distributed ledger infrastructure and user interface to collect and collate adverse event reports to create a comprehensive and interoperable database.

**Methods:**

A review of the literature will be conducted to identify the strengths and limitations of the current UK adverse event reporting system (the Yellow Card System). If insufficient information is found in this review, a survey will be created to collect data from system users. The results of these investigations will be incorporated into the development of a mobile and web app for adverse event reporting. A digital infrastructure will be built using distributed ledger technology to provide a means of linking reports with existing pharmaceutical tracking systems.

**Results:**

The key outputs of this project will be the development of a digital infrastructure, including a backend distributed ledger system and an app-based user interface.

**Conclusions:**

This infrastructure is expected to improve the accuracy and efficiency of adverse event reporting systems by enabling the monitoring of specific medicines or medical devices over their life course while protecting patients’ personal health data.

**International Registered Report Identifier (IRRID):**

PRR1-10.2196/28616

## Introduction

### Background

It is essential that adverse drug reactions and medical device events are reported to provide accurate and comprehensive warning labels and restrict or remove products that present too high of a risk to patient safety [1]. However, the number of adverse events reported in the United Kingdom has been declining [2]. Mitigating the barriers associated with spontaneous reporting systems and ensuring that those reports are useful are essential components of ensuring patient safety and reducing costs of post-market surveillance [3]. Therefore, this project aims to develop a distributed ledger infrastructure that will redesign the adverse event reporting and collecting process to improve patient care and safety.

Currently, the United Kingdom uses the Medicines and Healthcare products Regulatory Agency’s (MHRA) Yellow Card Scheme (YCS) for adverse event reporting. Reports submitted to the MHRA are stored in a database, in compliance with General Data Protection Regulation (GDPR) requirements [4,5]. Access to deidentified data can be requested under the Freedom of Information Act, but is otherwise only accessible to MHRA staff and, in some circumstances, health researchers. Confidential information is only made available if necessary to achieve specific health purposes, with conditions in place to protect patient information as best as possible [5].

The YCS has contributed to the identification of serious safety issues [6]; however, several usability issues with the public interface have been identified [7]. The YCS was redesigned in 2012 based on recommendations [7] and launched an app in 2015 to help make reporting easier [8]. An international Med Safety app has been developed (in collaboration from the MHRA) as part of the Innovative Medicines Initiative WEB-Recognising Adverse Drug Reactions (WEB-RADR) project [9] to provide a platform for members of the public to report adverse events. These reports are then transmitted to the relevant national databases [9,10]. In the United Kingdom, the WEB-RADR2 project is ongoing and aims to facilitate information sharing between health care and regulatory systems (by mapping terminologies and connecting with electronic health records) and to provide its features through application programming interfaces (APIs), tools that will eventually replace the Yellow Card website [11-13].

However, while the WEB-RADR2 project work packages include improving connectivity, their backend system (Vigilance Hub) only appears to allow the tailoring of the app to suit the needs of specific regulators (ie, to manage aesthetics, translations, reporting forms, news, lists of authorized products, and app users) [14]. Therefore, there is still a need for an innovative infrastructure to support the improvements made in the reporting interfaces by providing a means of verification. Without this infrastructure, the benefits of improved interfaces will not be fully realized.

Given the regulatory changes with Brexit, a decentralized system with simplified information sharing and consent procedures could have significant economic value. The potential benefit of blockchain for the medical supply chain, including pharmacovigilance specifically, has been recognized [15]. It could reduce the time and effort needed to trace medicines associated with adverse events (eg, to link adverse events reported in the United Kingdom with medicines produced in the European Union). A distributed ledger infrastructure would enable parties throughout Europe (and throughout the supply chain) to add information to the database that can then be made accessible to all other relevant parties, avoiding potential regulatory and data security issues that may occur.

There is a large and growing global pharmacovigilance market [16]. In 2019, spontaneous reporting — such as reports made to the YCS — had the biggest pharmacovigilance market share. It is a cost-effective means of detecting adverse events and is commonly used by both regulatory agencies and pharmaceutical companies [17]. Therefore, there is significant potential value in improving systems of spontaneous reporting and in improving the traceability, verification, and quality management of those data.

### Rationale

There is a need to improve confidence in shared data by considering provenance, traceability, verification, and quality management. The need is not to develop a new technology but to integrate existing technologies to develop a novel system to improve the specific challenge identified: the limitations and inefficiencies of the United Kingdom’s current YCS.

One of the key issues is a lack of reporting, which could be due in part to usability problems with the current system. The only previous usability study identified issues such as difficulties navigating and using the online form and overly complex language, which increased the effort required to submit a report [7]. The YCS was redesigned on the basis of this study, but no further usability evaluations were identified. For members of the public and health care professionals to report more adverse events, they need to understand how and what to report, be capable of using the reporting system, and be motivated to report. Taking full advantage of adverse event reports (AERs) that are submitted requires a more interoperable system that can verify the provenance of AER data by linking with the pharmaceutical and medical device supply chains.

This project has significant potential value to many stakeholders. Economically, there is value for manufacturing and pharmaceutical companies. The distributed ledger infrastructure would make it easier to track and manage individual drugs and devices, reducing costs of more labor-intensive tracking processes. It could also potentially help companies avoid costs associated with adverse events by identifying and addressing any harmful side effects of the drug or device earlier. In the longer term, the infrastructure could be used to identify and track causes of adverse events and pinpoint whether the problem was due to the drug itself or an issue during production, such as contamination, mixed labels, or an inaccurate amount of the active ingredient [18]. It could also support common manufacturing problems related to documentation [19]. This would help companies document issues and engage in continuous process improvement, which would also help avoid costs associated with adverse events.

An improved reporting system would also benefit patients, clinicians, and pharmacists by reducing the time and effort needed to submit AERs, which is expected to help increase the number of reports submitted. This offers significant public health value for patients; identifying and addressing harmful side effects earlier could reduce the number of people who will suffer from them. It also has the potential to improve patient empowerment by increasing the clarity and ease of reporting. If patients feel confident in reporting side effects that they consider unacceptable and demanding alternatives, this empowerment could help drive innovation in the pharmaceutical market. Additionally, it would help reduce the significant economic costs associated with adverse event–related hospitalizations for the UK National Health Service (NHS).

A more effective and efficient system for managing post-market surveillance data would have significant value for regulatory authorities and companies conducting their own post-market surveillance. It would help reduce costs associated with conducting post-market surveillance by reducing the effort needed to collect and compile all the relevant data. It could also provide new insights to inform guidance on the correct use of medicines and drugs.

There are also potential environmental benefits from making surveillance more efficient. Identifying and verifying problems with a medicine or medical device earlier could reduce the waste associated with producing and dispensing drugs that are ineffective or harmful.

### Aims and Objectives

This project will optimize the integration of the medicines and medical devices supply chain to provide a means of verifying the provenance, and improving the quality, of AERs made by UK patients and health care professionals. It aims to address 2 key problems with the current system: lack of reporting and lack of verification. The first problem will be solved by developing an easy-to-use app interface for adverse event reporting. This could replace the current data collection system but replicate the types of data collected. The second problem will be solved by developing an infrastructure that will integrate adverse event reporting with the entire pharmaceutical supply chain, enabling report provenance to be verified and associated with specific medical products. Together, these solutions will optimize the quality of data and analyses that can be derived from AERs to better prevent fraud and identify any potential health risks early.

This project will improve on the current adverse event reporting system by developing an infrastructure that supports interoperability, can integrate information from different sources to trace specific medicines and medical devices over their lifespan, and thus corroborate reports of adverse events and enable more rapid action to be taken when adverse events are identified. Our project will be more user-friendly and accessible than the YCS and will disrupt the existing systems (including YCS and WEB-RADR2) by decentralizing them to provide better information flow, increase security, and, crucially, allow for the provenance of AERs to be validated. The key objectives of this project are to identify the perceived benefits and barriers in the current reporting of adverse events by patients and health care providers and develop a distributed ledger infrastructure and user interface that can collect and collate AERs to create a comprehensive and interoperable database.

## Methods

### Study Design

The study will follow the Population, Intervention, Comparator, Outcomes (PICO) model (Table 1).

**Table 1:** Population, Intervention, Comparator, Outcomes (PICO) model.

Component	Description
Population	The primary target customers and end users chosen for this project are UK health care professionals (HCPs) and members of the public. They were chosen because barriers to the usability and effectiveness of the United Kingdom’s spontaneous reporting Yellow Card Scheme have been identified for both patients and health care professionals, including complex language, lack of feedback, lack of knowledge about the criteria for reporting, and lack of time to report [20-23].
Intervention	A new mobile and web app user interface will be developed, which is intended to increase user reporting of adverse events. A distributed ledger infrastructure will be developed to provide a means for regulatory agencies (like the MHRA^a^) to verify the provenance of the events reported to the app system by linking them to the medicines and medical devices supply chain.
Comparator	There is no comparator.
Outcomes	The outcomes are a prototype of the app and the distributed ledger infrastructure.

^a^MHRA: Medicines and Healthcare products Regulatory Agency.

### Data Collection

To address the first objective, a review of the literature will be conducted to identify specific benefits and barriers in the current reporting of adverse events by patients and health care providers in the United Kingdom. If insufficient information is found, a survey will be developed and conducted to collect user feedback about the current system of reporting.

To inform the development of the digital infrastructure, literature and reports relating to the current operation of the YCS will also be investigated. This will help to ensure that problems not identified by patients and health care providers are also detected and can be addressed in the design of the new system.

### Digital Infrastructure Development

A foundational level, based on distributed ledger technology, will form the infrastructure of the system, upon which applications can be built and provided. Farmatrust’s blockchain solutions have been used to provide multisite, multistakeholder, end-to-end supply chain tracing of pharmaceutical products for governmental, regulatory, hospital, and industry organizations. This experience and previous developmental work will provide a strong base on which to develop a similar end-to-end supply chain tracing system that links AERs with existing pharmaceutical tracking systems.

The current backend of the adverse event reporting system will be examined to identify inefficiencies. The core infrastructure will be developed using distributed ledger technology. The specific solution architecture to be used will be determined at this point, after further examination of the needs, inputs, and current limitations of the adverse event reporting system. A formal evaluation framework will be used to guide the development and ensure that the design choices fit the specific needs of the situation [24]. Interoperability with current and future databases and drug and medical device supply chain systems will be ensured by using the Fast Healthcare Interoperability Resources (FHIR) standard. This aspect of the project will also explore the options for governance processes to admit and remove actors from the system.

### User Interface Development

Alongside the development of the system infrastructure, a mobile and web app will be developed to provide an easy-to-use and generally accessible interface for patients and health care providers to report adverse events to the system. This development will account for benefits and barriers identified in the literature and from user research.

### Patient and Public Involvement

In line with the principles of user-centered design [25,26], members of the public and health care professionals will be recruited to collaborate on the development of the adverse event reporting app, to ensure that it is user-friendly and addresses their needs.

### Ethics and Dissemination

The infrastructure will be built to comply with all GDPR and the British Data Protection Act (DPA 2018) requirements [27,28]. Potential ethical issues relating to the development of a digital infrastructure to record and verify personal and sensitive medical information will be identified from the literature and in discussions with the public representatives.

If user research is conducted, a participant information sheet will be produced and provided to participants upon recruitment before getting their informed consent. This will explain the study and detail their rights with respect to withdrawing and having their data removed. Data protection procedures will also be established and made clear.

A paper detailing the methods and results of this project will be submitted to academic peer-reviewed journals, conferences, and clinical meetings. The public representatives will also be consulted about the dissemination of the results of the study to the public in generally accessible formats.

## Results

The key output of this project will be the development of a digital infrastructure for a new system of adverse event reporting, including a backend distributed ledger system and a mobile and web app user interface. The novel user interface will help to address the challenge of increasing adverse event reporting by patients and health care professionals. The distributed ledger system will provide a means of verifying this information using zero knowledge proof by tracking the medicine or medical device in question to confirm that the correct item was delivered to and used by the intended recipient for the correct purpose.

## Discussion

The infrastructure developed will be able to use zero knowledge proofs to verify the provenance of AERs and reduce risks to patient confidentiality and data privacy. While data security and privacy concerns can never be completely avoided, these methods should provide a more secure system for confidential patient data. The decentralized nature of the system means that specific medicines or medical devices can be traced over their life course and reported adverse events can be linked to the specific product without compromising the patient’s personal health data. This means that individual AERs can be verified by checking that the medicine or device in question has been dispensed and to the intended recipient. The traceability of specific products enables the system to highlight any discrepancies in the AER. A more user-friendly interface and a means of verifying reports are expected to increase ease of use, efficiency, and the quality and quantity of adverse event data reported.

### Anticipated Impact

Estimates of the cost of preventable drug-related adverse events in the United Kingdom are in the range of £100-400 million a year [29-31]. A digital system that operates more efficiently, operates with lower costs, and increases the number of adverse events reported could save money by identifying problems earlier. A more efficient system that identifies and verifies adverse events earlier could also reduce waste. By reducing inefficiencies (ie, reducing the labor-intensive management of the existing adverse event reporting system), production of the suspect drugs or devices could be paused earlier to await investigation. This would avoid the production and shipping of many drugs or devices that would otherwise have been deployed and potentially recalled, reducing waste. The economic savings for pharmaceutical and manufacturing companies could free up money for increased investment in industrial digitalization research, as less money would be needed to deal with adverse events.

In addition to economic savings and reduced waste of resources, the proposed distributed ledger infrastructure will be open, interoperable, and able to provide zero knowledge proof, thus reducing concerns about data privacy and security. As patients are encouraged to report all side effects, even known ones [32], to enable more accurate understanding of the prevalence of side effects of drugs, this system will have the potential to capture a very large amount of real-world data. The openness, access, and accountability of the infrastructure will be a mechanism to enable collaborations and interaction between academia, health care systems, the established manufacturing sector, and smaller, start-up digital technology companies.

### Risks

Given the level of innovation, the details of technical implementation will depend on factors such as interoperability gateway configurations, computational capacities required, and adoption by the end users. This risk will be mitigated by a development approach that will use the FHIR standard and user research to identify needs and barriers.

There are also potential risks from a health care perspective. Health care providers may be unwilling to report if concerned about disciplinary action; however, an anonymous and easy-to-use system should enable reporting to be more easily incorporated into routines in health care environments without personal risk. Flaws in the system could potentially expose patients to higher risk medicines and medical products, mitigated by careful design and testing before promoting for implementation and the use of zero knowledge proof encryption.

### Future Directions

In this project, the infrastructure developed will be designed to replace the current YCS operated by the MHRA. The main barrier to implementing our distributed ledger infrastructure is whether the MHRA will accept the change in system. To verify the provenance of AERs, it will be necessary to link the reports with the pharmaceutical supply chain. Because barcode serialization technology is already used to label and track individual packets of medicine or medical devices [33], the Farmatrust system will provide the technology to link AERs with the serialized medicine or device. However, to do this, access will have to be granted via a wholesaler who can access the National Medicines Verification System (NMVS, or SecurMed in the United Kingdom) database that tracks the pharmaceutical supply chain [34].

Future studies will be needed to test the feasibility, usability, and efficacy of the system. As the front-end and back-end components could theoretically be implemented separately, separate evaluations could provide useful information on their independent impacts on adverse event reporting and pharmacovigilance. In the longer term, this project could easily be expanded and adapted for global use. It could provide a means of linking data across countries. Applications could be added to address the needs of specific stakeholders (eg, pharmaceutical and manufacturing companies) to identify relevant issues from the AERs and enable continuous improvements in products and processes.

There are many applications — beyond verifying AERs — that our infrastructure could support. For instance, because the system links AERs with the pharmaceutical supply chain, it could be used to trace individual packets of medicine or medical devices. This could be very valuable in the case of recalls — specific packets or devices could be tracked, and information about their distribution and dispensation almost instantly provided. Alerts could then be sent to wholesalers and recipients of the medicines or devices. The number of potential applications provide an opportunity for long-term growth and productivity, and these applications (eg, the particular method used to check the report with the supply chain database and create a report) can be protected with patents. After the feasibility study, we will aim to market this solution globally.

## Abbreviations

AER: adverse event report
API: application programming interface
DPA: Data Protection Act
FHIR: Fast Healthcare Interoperability Resources
GDPR: General Data Protection Regulation
MHRA: Medicines and Healthcare products Regulatory Agency
NHS: National Health Service
NMVS: National Medicines Verification System
WEB-RADR: WEB-Recognising Adverse Drug Reactions
YCS: Yellow Card Scheme
